# Parameters associated with radiographic distal surface caries in the mandibular second molar adjacent to an impacted third molar

**DOI:** 10.1186/s12903-023-02766-w

**Published:** 2023-02-24

**Authors:** Verena Toedtling, Tim Forouzanfar, Henk S. Brand

**Affiliations:** 1 Department of Oral and Maxillofacial Surgery, Faculty of Dentistry, Academic Centre for Dentistry Amsterdam (ACTA), University of Amsterdam and VU University Amsterdam, Gustav Mahlerlaan 3004, 1081 LA Amsterdam, The Netherlands; 2 Department of Oral Biochemistry, Faculty of Dentistry, Academic Centre for Dentistry Amsterdam (ACTA), University of Amsterdam and VU University Amsterdam, Gustav Mahlerlaan 3004, 1081 LA Amsterdam, The Netherlands

**Keywords:** Surgery, Dental disease, Public health, Prevention, Distal surface caries, Third molar, Second molar, Mandibular molar, Proportion, Risk

## Abstract

**Background:**

To determine the risk factors for the development of radiographic distal surface caries (rDSC) in patients who attend routine dental check-ups during an era of National Institute for Health Care Excellence third molar surgery guidelines.

**Methods:**

Radiographs taken during routine dental examinations involving 1012 patients from Manchester, UK were accessed. Clinical parameters, oral health, patient demographics, and socioeconomic factors were assessed. Risk factors were identified by multivariate logistic regression analysis.

**Results:**

The detected rate of rDSC was 63.9% and rDSC was distributed homogenously across all five socioeconomic groups (*p* = 0.425). Risk factors associated with rDSC (*p* < 0.001) were identified as partially erupted mesio-angularly impacted mandibular third molars, third molars with compromised molar to molar contact points, loss of lamina dura of ≥ 2 mm, male gender, increasing age, and a higher modified Decayed Missing Filled Tooth score.

**Conclusion:**

rDSC was significantly associated with the angulation of third molars, the compromised contact position of the adjacent third molar, the periodontal status of the distal aspect of the second molar and the cumulative history of oral health in a population governed by specific third molar guidelines. An active approach to third molar surgical management could reduce rDSC and serve this population, irrespective of patients’ socioeconomic or deprivation status.

## Introduction

In the UK, clinical guidelines state that impacted lower third molars should be left in situ unless strict criteria are met. Reasons for removal, as stated in the National Institute for Health Care Excellence (NICE) guidance 2000, are repeated episodes of pericoronitis, unrestorable caries, non-treatable pulpal or periapical pathology, abscess, osteomyelitis, internal or external resorption, fracture, tooth impeding surgery, reconstructive jaw surgery, tooth involved in tumour resection, cellulitis or disease of the follicle including cysts/tumours [1]. However, clinicians and recent studies draw attention to an increasing detection rate of caries that develop in the distal aspect of the lower second molar teeth as a result of the persistence of food and debris stagnation and inaccessibility to cleaning devices leading to mature dental plaque between both teeth. An in-depth analysis of the literature found a significant proportion of distal surface caries (DSC) in mandibular second molars. However, the risk was usually assessed in referred hospital patients and does not relate to the risk in the general dental population [2]. We foresee a difference in the proportion of DSC between UK hospital referred populations and patients who are routinely assessed and, as a consequence, a difference in the pattern of dental disease associated with impacted mandibular third molars and its risk factors. We also hypothesised that patients in the general population with impacted third molars have a greater dental caries experience in the adjacent second mandibular molar when they belong to areas of lower socioeconomic status. Therefore, this retrospective observational study aimed to determine the rate of distal surface caries detected on radiographs (rDSC) in a non-third molar assessment-based population guided by strict third molar removal indications. The study sought to correlate this with potential risk factors such as the orientation and contact point localisation of the adjacent third molar, periodontal support of the second molar, patient demographics, past dental disease experience and socioeconomic status.

## Materials and methods

The investigator (VT) designed and implemented a retrospective cross-sectional study which was submitted via the Integrated Research Application System (IRAS) (ref: 265014). Ethical approval was obtained from the Health Research Authority (HRA), Health and Care Research Wales (HCRW) (ref: 20WM/0008), West Midlands—Solihull Research Ethics Committee and the Confidentially Advisory Group (CAG) London Committee (ref: 20/CAG/0050). The study was conducted in full conformance with all the relevant legal requirements and the principles of the Declaration of Helsinki, Good Clinical Practice (GCP) and the UK Policy Framework for Health and Social Care Research 2017.

The sample size for this study was calculated by power calculation (G*Power 3.1.5, Heinrich-Heine-Universität Düsseldorf), which allowed for a CI of 95% with a 5% margin of error (standard power level of 80% and alpha level of 80%, *p* = 0.05). A sample size of 969 was calculated and 1012 patients were assessed. The retrospective study samples were derived from populations of patients who presented to the Manchester University NHS Foundation Trust, University Dental Hospital, UK and who attended routine examinations and had bitewings and periapical radiographs taken. In addition, the investigator had access to a panoramic radiograph of some of the included patients. All patients had been managed and treated in the past by the local dental team and the target population of this study included adults who attended a dental appointment for caries screening within a national healthcare-based setting. The investigator of the centre accessed the previously taken radiographs electronically via a Picture Archiving and Communication System (PACS). This medical imaging technology was used to perform a re-evaluation of specific patient characteristics. Included were radiographs taken after 31^st^ January 2012 of patients ≥ 25 years of age with impacted and partially erupted mandibular third molars adjacent to a second mandibular molar. Fully erupted and functional third molars were excluded from the study. We only included excellent quality images (Grade 1). Images with artefacts such as severe cervical burnout and positioning errors or otherwise obscured areas of interest were excluded. We also excluded second molars with full coverage crowns or which were extensively restored or with severe decay or retained roots. However, when both the right and left sides of the same patient met the inclusion criteria, only one image was randomly selected by tossing a coin. All images with a head outcome were included in the study. This resulted in a final study population of 1012 patients.

**Fig. 1:**
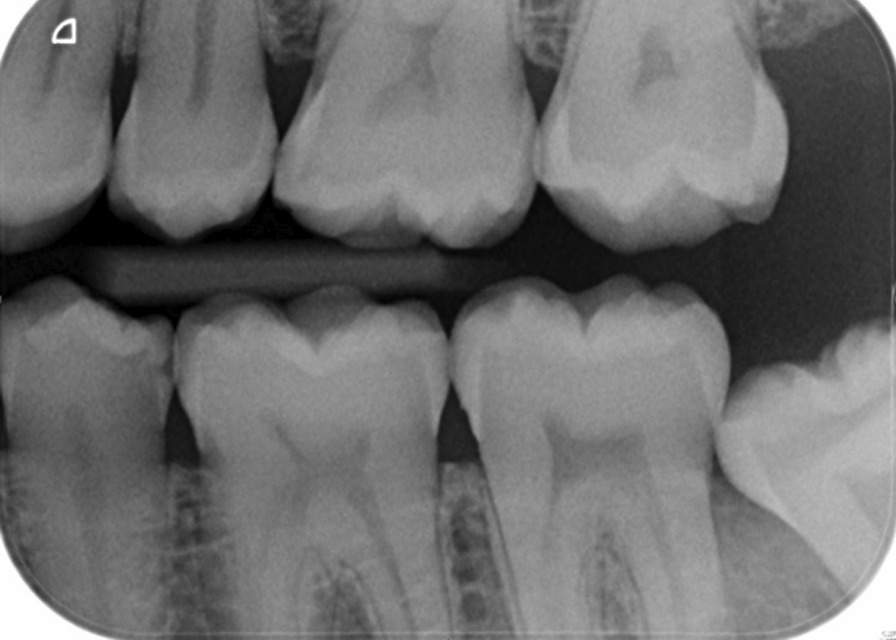
Left bitewing radiograph of impacted and partially erupted mandibular third molar contacting the adjacent second molar below the CEJ

**Fig. 2:**
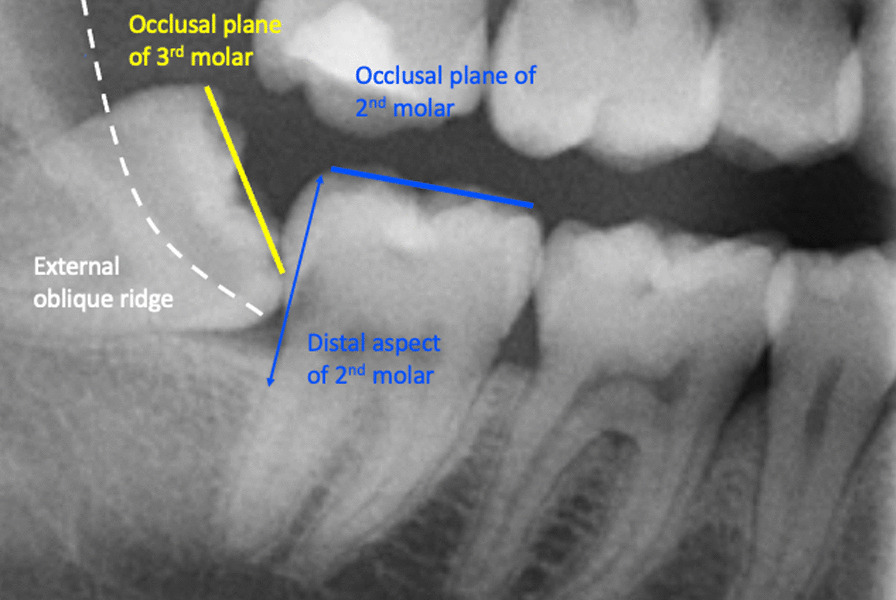
Indication of various anatomical points required for the assessment of third molar partial emergence/eruption on a panorama radiograph

In this study, partial eruption or partial emergence of the mandibular third molar was determined by assessing the third molar position in relation to the adjacent second molar and the anatomical landmarks. The third molars were deemed to be partially erupted when one of the third molar cusps was positioned above the external oblique ridge or the occlusal plane level of the neighbouring second molar. In cases where these anatomical landmarks could not be assessed on the radiograph, the Cementoenamel Junction (CEJ) of the adjacent mandibular second molar in relation to the marginal ridge position of the adjacent third molar was also used to obtain information on the eruption status of mandibular third molars and its depth of impaction. This assessment method is a modified description of the Pell & Gregory classification and class I B, class II B, class III A and B from the original Pell & Gregory categorisation were included [3]. This was applied to all third molar angulation types (mesial, distal, vertical, horizontal and transverse). Figure 1 illustrates an impacted and partially erupted mandibular third molar on a bitewing radiograph contacting the second mandibular molar below the CEJ. Figure 2 shows a section of a panoramic radiograph of a mandibular third molar that was deemed partially erupted. The external oblique ridge or bony anterior border of the ramus that appears radiopaque and is located on the outer aspect of the mandible which runs from the ramus to the first molar is indicated with a white dashed line.

The primary outcome of interest was caries on the distal aspect of the mandibular second molar (rDSC). A caries lesion was determined to be present when a radiolucency with irregular morphology and margins could be detected in enamel and/or dentine or cementum. The secondary outcomes of interest included variables such as patient demographics, socioeconomic status and oral health status. The study investigator of the centre collected the following information: patient ID, age (in years) and gender. They also collected the following radiographic characteristics: side of the mandible (right or left), angulation type of the third molar according to Winter’s classification, periodontal status assessed by recording the vertical lamina dura (LD) loss in millimetres distally to the second mandibular molar, and the mesial-buccal cusp relationship of the third molar with the CEJ of the adjacent second molar (molar-to-molar contact point: above, below, at or no contact with the CEJ of the adjacent second molar). These data were recorded on standardised Excel spreadsheets. We also adapted the traditional Decayed, Missing, Filled Tooth (DMFT) index for use in this study according to the radiographic appearance of the total number of decayed, missing and filled teeth on radiographs. This is a modified version (mDMFT-R) of the original DMFT index. The formula we developed to calculate the mDMFT-R score was: DMFT count × 100 / tooth count (tooth crowns fully visible on the radiograph). To assess the socioeconomic status of the patients from Manchester, the postcode of their home address was entered into an online conversion tool and was adapted to the Index of Multiple Deprivation (IMD) score which has five categories. The ranges of IMD scores from least to most derived were: Category 1, ≤ 8.49; Category 2, 8.5–13.79; Category 3, 13.8–21.35; Category 4, 21.36–34.17; Category 5, ≥ 34.18 [4].

A pre-data collection test was performed to provide feedback on the protocol. Calibration meetings were held via Zoom, and assessments of standards and data collection methods and approaches were standardised. A pilot study was performed before the full study to assess the work and data collection flow. The radiographs were initially viewed and assessed for mandibular second molar characteristics, such as the presence of rDSC and LD loss of ≥ 2 mm, by one observer. Subsequently, the entire data set was reassessed by a second observer and any disagreements were resolved by consensus. To analyse the intra-observer agreement, 102 randomly selected cases (10% of the whole study population) were reassessed by the two observers after at least six weeks.

The statistical analysis was performed using IBM SPSS Statistics Version 26.0 for MAC (Release 26.0.0.0, 64-bit edition). The intra-observer and inter-observer agreement on the radiographic finding of rDSC and loss of LD was analysed using the Cohen’s *K* test (an agreement of 0.75 to 1.00 was considered excellent, 0.60 to 0.74 good, 0.40 to 0.59 moderate, and less than 0.40 poor). The associations between rDSC in the second mandibular molar and radiographic, demographic, socioeconomic and oral health variables were analysed using Pearson’s chi-square test and the t-test was used to compare the means of the two groups. Additionally, a multivariate logistic regression model was used to further evaluate predictive values for the risk factors of the rate of rDSC. The significance level was set at *p* < 0.05.

## Results

On PACS, 8304 patients were viewed and 1012 (12.18%) patients were included. The level of agreement between the two observers for rDSC and loss of LD was excellent (*κ* = 0.776, *p* < 0.0001). The intra-observer reliability of both observers was also excellent (*κ* of observer A = 0.812, *κ* of observer B = 0.797; *p* < 0.0001). Six hundred forty-seven of the included 1012 patients were affected, resulting in a rate of rDSC of 63.9%. Fifty-three percent (n = 532) of third molars were located on the left side of the mandible and 57% (n = 578) of the affected patients were male. The percentages in each age (years) category were as follows: 25–30 (39.4%, n = 402), 31–35 (20.3%, n = 205), 36–40 (11.4%, n = 115), 41–50 (16.4%, n = 166), 51–60 (8.2%, n = 83) and ≥ 61 (4.1%, n = 41). The orientations of the third molars were: mesial (59.2%, n = 599), horizontal (20.3%, n = 203), vertical (9.7%, n = 98), distal (10.8%, n = 109) and transverse (0.3%, n = 3). The molar-to-molar contact points of the mesial-buccal cusp position of the third molar in relation to the CEJ of the second molar were: above (2.6%, n = 26), at (10.9%, n = 110), below (84.6%, n = 846) and no contact (3.0%, n = 30). In this patient sample, 13.6% (n = 138) had < 2 mm loss of LD and 86.4% (n = 874) had ≥ 2 mm loss of LD on the distal aspect of the second molar. The IMD quintile scores were (from 1 least deprived to 5 most deprived): 1 (11.3%, n = 114), 2 (12.7%, n = 129), 3 (16.4%, n = 166), 4 (21.1%, n = 214), 5 (36.1%, n = 365) and missing score (2.4%, n = 24). The mDMFT-R (%) group scores were as follows: 0 (7.2%, n = 73), 1–15 (7.9%, n = 80), 16–30 (17.2%, n = 178), 31–45 (17.7%, n = 179), 46–60 (16.6%, n = 167), 61–75 (17.1%, n = 173) and ≥ 76 (16.4%, n = 166).

The presence of rDSC in the second molar and its association with various patient demographics, radiographic characteristics and socioeconomic and oral health status are shown in Table 1. Pearson’s chi-square independence test indicated that the variables were significantly associated with the occurrence of rDSC in second mandibular molars, with the exceptions of the side of the mandible and socioeconomic status. Among the different groups of angulations of mandibular third molars, mesially inclined were related to the highest proportion of rDSC in the second mandibular molar (78.3%), followed by horizontally inclined third molars (55.7%). rDSC was less frequently observed in patients in which the molar-to-molar contact was above the CEJ (24.6%), compared to those patients in which the contacts were made at or below the CEJ (27.3% and 70.8% respectively). There was a statistically significant increase in rDSC with increasing age (*p* < 0.001). rDSC was also significantly more frequently observed in patients with loss of LD of ≥ 2 mm (71.4%) and increasing mDMFT-R percentages. Male patients were significantly more frequently affected by rDSC (68.2%) than female patients (58.3%). Further analysis showed that when comparing male (n = 394) and female (n = 253) patients with rDSC they had identical mean ages (38 years) and loss of LD ≥ 2 mm (96.6%), equal mean IMD quintile groups (Category 4), and very similar mean mDMFT-R scores (51.6% and 52.1% respectively). The most common cusp position was below the CEJ (92.9% and 92.1% respectively) and the most frequent third molar orientation was mesially angulated (71.6% and 73.9% respectively). Both gender groups were not significantly different from each other.

**Table 1:** Detected rate of rDSC in mandibular second molars adjacent to impacted third molars and its relation to clinical, demographic, socioeconomic and oral health characteristics

Characteristic	Total	Presence of rDSC		*p-*value
	*n* = 1012 (%)	Yes (%)	No (%)	
Side of mandible				0.883
Right	480 (47.4)	308 (64.2)	172 (35.8)	
Left	532 (52.6)	339 (63.7)	193 (36.3)	
Gender				0.001*
Female	434 (42.9)	253 (58.3)	181 (41.7)	
Male	578 (57.1)	394 (68.2)	184 (31.8)	
Mean age (years) ± SD	36.6 ± 11.1	38.1 ± 11.9	33.9 ± 8.9	< 0.001*
Age (years)				< 0.001*
25–30	402 (39.4)	236 (58.7)	166 (41.3)	
31–35	205 (20.3)	105 (51.2)	100 (48.8)	
36–40	115 (11.4)	80 (69.6)	35 (30.4)	
41–50	166 (16.4)	126 (75.9)	40 (24.1)	
51–60	83 (8.2)	64 (77.1)	19 (22.9)	
≥ 61	41 (4.1)	36 (87.8)	5 (12.2)	
Orientation of third molar impaction				< 0.001*
Mesial	599 (59.2)	469 (78.3)	130 (21.7)	
Horizontal	203 (20.1)	113 (55.7)	90 (44.3)	
Vertical	98 (9.7)	38 (38.8)	60 (61.2)	
Distal	109 (10.8)	25 (22.9)	84 (77.1)	
Transverse	3 (0.3)	2 (66.7)	1 (33.3)	
Contact point localisation: MB cusp position				< 0.001*
Above	26 (2.6)	9 (24.6)	17 (65.4)	
At	110 (10.9)	30 (27.3)	80 (72.7)	
Below	846 (83.6)	599 (70.8)	247 (29.2)	
No contact	30 (3.0)	9 (30)	21 (70)	
Loss of lamina dura				< 0.001*
< 2 mm	138 (13.6)	23 (16.7)	115 (83.3)	
≥ 2 mm	874 (86.4)	624 (71.4)	250 (28.6)	
Mean quintile group IMD score ± SD	3.6 ± 1.4	3.7 ± 1.4	3.5 ± 1.4	0.280
Quintile group IMD score				0.425
1 (least deprived)	114 (11.3)	68 (59.6)	46 (40.4)	
2	129 (12.7)	81 (62.8)	48 (37.2)	
3	166 (16.4)	98 (59.0)	68 (41.0)	
4	214 (21.1)	142 (66.4)	72 (33.6)	
5 (most deprived)	365 (36.1)	244 (66.8)	121 (33.2)	
Missing	24 (2.4)	14 (58.3)	10 (41.7)	
Mean mDMFT-R (%) ± SD	47.5 ± 28.2	51.8 ± 27.7	39.9 ± 27.6	< 0.001*
mDMFT-R (%)				< 0.001*
0	73 (7.2)	22 (30.1)	51 (69.9)	
1–15	80 (7.9)	40 (50.0)	40 (50.0)	
16–30	174 (17.2)	111 (63.8)	63 (36.2)	
31–45	179 (17.7)	118 (65.9)	61 (43.1)	
46–60	167 (16.6)	115 (68.9)	52 (31.1)	
61–75	173 (17.1)	107 (61.8)	66 (38.2)	
≥ 76	166 (16.4)	134 (80.7)	32 (19.3)	

MB cusp, Mesial-Buccal cusp; IMD, Index of Multiple Deprivation; mDMFT-R (modified decayed, missing, filled, tooth index applied to radiographs). * Statistically significant (*p* < 0.05). Pearson’s chi-square independence test performed between categorical variables and the t-test to test between means of two groups

The multivariate logistic regression analysis (Table 2) revealed the following risk factors for developing rDSC: third molars with mesial angulation (OR = 3.62, *p* ≤ 0.001), loss of LD of ≥ 2 mm (OR = 6.55, *p* ≤ 0.001), molar-to-molar contact points below the CEJ (OR = 4.21, *p* = 0.002), male gender (OR = 1.51, *p* = 0.010) and patients with ages between 41 and 50 years (OR = 2.20, *p* = 0.002). Also, all mDMFT-R scores had a statistically significantly greater odd of rDSC in comparison to the reference mDMFT-R score (OR = 2.57–6.10; *p* = 0.015—*p* < 0.001).

**Table 2:** Multivariate logistic regression model for rDSC

	OR	95% CI of OR		*p-value*	Coefficient
		Lower	Upper		
Gender					
Female	1				
Male	1.51	1.11	2.10	0.010*	0.42
Age (years)				< 0.001*	
25–30	1				
31–35	0.55	0.37	0.83	0.004*	- 0.59
36–40	1.51	0.89	2.55	0.124	0.41
41–50	2.20	1.34	3.62	0.002*	0.79
51–60	1.66	0.86	3.20	0.132	0.51
≥ 61	2.85	0.96	8.44	0.060	1.05
Orientation of third molar impaction				< 0.001*	
Vertical	1				
Distal	0.63	0.30	1.32	0.221	- 0.46
Horizontal	1.36	0.71	2.59	0.352	0.31
Mesial	3.62	1.98	6.59	< 0.001*	1.29
Transverse	6.36	0.36	111.74	0.206	1.85
Contact point localisation: MB cusp position				0.021*	
No contact	1				
Above	5.36	1.30	22.16	0.020*	1.68
At	3.67	1.21	11.13	0.021*	1.30
Below	4.21	1.67	10.59	0.002*	1.44
Loss of lamina dura					
< 2 mm	1				
≥ 2 mm	6.55	3.71	11.60	< 0.001*	1.88
mDMFT-R (%)				< 0.001*	
0	1				
1–15	2.57	1.20	5.51	< 0.015*	0.95
16–30	3.72	1.91	7.25	0.001*	1.32
31–45	3.37	1.74	6.55	0.001*	1.22
46–60	3.77	1.93	7.40	0.001*	1.33
61–75	2.49	1.27	4.87	0.008*	0.91
≥ 75	6.10	2.93	12.70	0.001*	1.81

1 Reference group; OR, Odds ratio; CI, Confidence interval. * Statistically significant (*p* < 0.05) by multivariate logistic regression analysis

## Discussion

The literature suggested that third molar retention over the long-term results in oral detriment and that impacted third molars cause plaque retention leading to caries on the distal aspect of the second molar [5]. However, the cariogenic risk factors of rDSC are still currently unknown. Our study assessed the rate of rDSC in the second mandibular molar and IMD status of patients who had intra-oral radiographs taken as part of routine dental check-ups, during a period when the NICE third molar surgery guidelines were issued and strictly followed in the UK. Strict adherence to the guidelines was ensured by regular trust wide audits of patients records of reasons for third molar removal as well as trust referral policy on third molar removal which were in place. Outcomes of the assessments and audits were regularly presented during four annually timetabled trust clinical effectiveness meeting days and the data were subsequently submitted to insurance providers with the aim to show evidence of compliance thus reduce the insurance fee for the hospital. In our study, we aimed to determine the risk factors for the development of rDSC in this patient population during this era.

The present study found a higher proportion of rDSC (63.9%) than previous studies which reported a rate of up to 52% [6]. A systematic review with a meta-analysis of previous studies on patients who underwent preoperative assessment for the removal of third molars reported a proportion of one in five patients [2]. Another study investigated the proportion of non-third molar assessed populations and reported a rate of 31.6% by examining CBCT scans [7]. Other studies reported distal caries on panoramic radiographs, which ranged from 4.35 to 38% [8, 9]. The lower rate in these previous studies may be because most studies solely used panoramic radiographs to detect rDSC, and extra-oral radiographs are much less sensitive in caries detection than intra-oral radiographs [10]. The high rate of rDSC in the present study might also be related to the strict inclusion criteria used. It is well documented that third molar crown completion and eruption are usually around 12–16 and 16–21 years old respectively. However, there is wide individual variation in eruption times and root formation of the third mandibular molar is usually completed around 18–25 years old, limiting positional changes of the third molar [11, 12]. Research suggests that interproximal caries take around two to three years to develop [13] and there is some evidence that rDSC peaks around 32 years of age in a population referred for third molar assessment [14]. We included female and male patients aged ≥ 25 years of age to ensure that third molar root formation was completed. In contrast, in previous studies, populations as young as 16–22 years of age were assessed for DSC and, consequently, DSC may have been more difficult to detect [15]. A further reason why the present study found a higher rDSC rate could be that we only included partially erupted and superficial impaction third molars as we aimed to assess this specific clinical relationship. This set a baseline that the third molar was in communication with the oral cavity and differentiated partially erupted and impacted third molars from unerupted impacted and functional third molars, as second molars adjacent to both unerupted and functional third molars have a reduced risk of DSC [16]. However, since the eruption of a tooth is a clinical parameter, the partial eruption of the mandibular third molar in the present study was radiographically determined by meticulously assessing its position in relation to the adjacent second molar and anatomical landmarks. However, the most likely explanation for the high detection rate of rDSC in the present study is that the data were collected after the introduction of the NICE third molar clinical guidelines in England, United Kingdom (UK) in 2000. This ensured that all radiographs included in the present study were taken when strict third molar removal criteria were in force and clinical audits were performed regularly to ensure compliance of clinicians to these guidelines. Whilst UK-based clinicians strictly adhered to the NICE guidelines and only removed symptomatic third molars or when specific pathology was present, many international clinicians do not face such restrictive guidelines but can discuss the risk of third molar retention with their patients and offer a patient-tailored-approach [17–23]. As a result, clinicians outside the UK might be more likely to remove third molars, with a subsequent lower rate of rDSC.

One main characteristic associated with rDSC is the angulation of the adjacent third molar. The literature repeatedly describes a strong association with mesially impacted third molars adjacent to impacted third molars [24–26]. This is in line with the present study where multivariate logistic regression analysis (Table 2) revealed that third molars with mesial angulation have a significantly greater probability of rDSC in the second molar (OR = 3.62, *p* ≤ 0.001). Another anatomical variation that is significantly associated with rDSC is the molar-to-molar contact points region and contacts below the CEJ are at 4.21 times greater risk of rDSC compared to third molars with no contact with the adjacent molar. There has been much speculation about the molar-to-molar contact point but very few studies have examined this explicitly [7, 27]. The American Association of Oral and Maxillofacial Surgeons (AAOMS) states in their white paper that when third molars are impacted and have an uncharacteristic molar-to-molar contact limiting the third molar’s functional ability, the third molar is classified as pathological, and this justified early surgical removal in the US [28]. Our regression analysis showed that all atypical contact points between mandibular second and third molars are associated with rDSC although molar-to-molar below the contact points are most strongly linked to rDSC (*p* = 0.002). We believe that rDSC localisation can be categorised into the following groups: rDSC above the usual molar-to-molar contact is a form of smooth surface caries, rDSC at the usual molar-to-molar contact is a form of interproximal caries and rDSC below the usual molar-to-molar contact is a type of root surface caries. No direct contact between molars may be compared to a situation where a third molar is absent. In this study, the various types contribute to the overall rate of rDSC (63.9%): 0.6%, above, smooth surface; 3%, at, interproximal; 59.3%, below, root surface; and 3%, no contact, absent third molar. This indicated that in the vast majority of patients, rDSC affects the root surface of the second molar next to mesially and horizontally angulated third molars.

We radiographically examined the loss of LD to assess the loss of periodontium. An intact LD is considered a sign of a healthy periodontium [29]. Few studies have previously studied the relationship between these two parameters and according to our knowledge, this is the first study investigating the relationship between the two measurements using linear regression analysis of rDSC. We found that third molars with radiographic evidence of loss of LD ≥ 2 mm on the distal aspect of the second molar were 6.55 times more likely to be associated with rDSC compared to the third molar with loss of LD < 2 mm. This finding was highly statistically significant and was the variable in the multivariate log regression analysis with the highest odd ratio and a positive coefficient (1.88). Consequently, we believe that vertical loss of LD of ≥ 2 mm is a significant precursory state and predictor for the projection of rDSC on the root aspect. From our observations, we also found that rDSC takes place on the root aspect after the occurrence of LD loss, allowing access to the exposed root surface of the distal aspect of the second molar. Thus, we propose that loss of LD ≥ 2 mm is an important precursor that may be used to predict rDSC risk in susceptible patients, especially those with other risk factors. The literature suggested that rDSC peaks during the early fourth decade of life in a third molar assessed population. However, we found in a population who attended for dental check-up that the rDSC group had a mean age of 38 years while the rDSC free group was on average four years younger. Further analysis showed that the average age of patients with rDSC and mesially and horizontally angulated third molars was lower compared to rDSC patients with distal, vertical and transverse third molars. This suggests that mesial and horizontal third molar angulations may have greater cariogenic potential. Therefore, it would be interesting to compare the microbiological profile of dental plaque and the relative abundance of each microbe from the distal surface of the second mandibular molar adjacent to different third molar angulations.

In 2019, McArdle and McDonald reported lower DMFT scores of patients with DSC than those of a surveyed population [30]. In the present study, the mean modified mDMFT-R was used, which was significantly higher in patients with rDSC in comparison to rDSC-free patients. Nonetheless, the clinical relevance of this observation seems limited. Firstly, the relatively small difference in mDMFT-R (< 12%) would be difficult to clinically differentiate and secondly, the patients with rDSC were on average four years older, and caries experience and resultant DMFT score increase with age. In the present study, male patients had a 1.51 times greater probability of suffering from rDSC in comparison to female patients. When we performed a further analysis, we could not find a significant difference in the sample characteristics of both genders that could explain the observed rDSC risk. However, it has been documented in the literature that men visit dentists less frequently compared to women. Men seek oral treatment more often for acute dental problems rather than chronic conditions and less frequently for disease prevention [31]. It has also been documented that women exhibit more positive attitudes about dental visits, greater oral health literacy and better oral health behaviours [32]. Thus, women may be more likely to have their third molar-related pathoses treated, with eventual third molar removal surgery and consequently are at reduced risk of rDSC. Interestingly, the proportion of rDSC within the investigated patient population showed that rDSC was equally distributed across all IMD groups, therefore affecting the least deprived almost as frequently as the most deprived patients. Since there is a well documented strong association of socioeconomic status with general health and oral health, including caries and periodontal disease [33] a similar increase in rDSC in more deprived categories was expected. The lack of a relation between rDSC and socioeconomic status in the present study can be explained by the strict clinical third molar removal NICE guidelines, which are applied regardless of a person’s socioeconomic status. The present study has a number of potential limitations. First, no attempt was made to assess the severity of the lesions. Future research on this topic should include the depth of caries lesions and explore whether both enamel and dentine are involved. In addition, our study could have been improved by clinical verification as both clinical and radiographic examinations should be performed for increased accuracy of diagnosis. On the other hand, the advantages of the present study are the large sample size and the use of a multivariate analysis. This permits analysis of more than one independent variable that influences the outcome variable, leading to more accurate results.

## Conclusion

rDSC in the second mandibular molar adjacent to a partially erupted and impacted third molar is a common clinical condition with a high rate in all socioeconomic groups in a population bound by specific third molar removal indications and guidelines. Long-term retention of third molars as well as clinical characteristics such as mesial impaction, compromised contact points and loss of LD of ≥ 2 mm are associated with increased risk of rDSC and almost exclusively affect the root aspect of the second molar. Future studies in well-selected study populations of nations with preventative third molar removal could provide evidence of whether preventative third molar removal has the potential to prevent rDSC in second mandibular molars adjacent to impacted and partially erupted third molars. Loss of LD ≥ 2 mm on the distal aspect of the second molar in the presence of an impacted and partially erupted mandibular third molar may present a newly described risk indicator for rDSC.

## Data Availability

The data that support the findings of this study are available on request from the corresponding author. The data are not publicly available due to privacy or ethical restrictions.

## Abbreviations

AAP: American Academy of Periodontology
AAOMS: American Association of Oral and Maxillofacial Surgeons
CEJ: Cementoenamel junction
CAG: Confidentially Advisory Group
DMFT: Decayed, missing, filled tooth
DSC: Distal surface caries
rDSC: Radiographic distal surface caries
GCP: Good clinical practice
HRA: Health Research Authority
HCRW: Health and Care Research Wales
IMD: Index of multiple deprivation
IRAS: Integrated Research Application System
LD: Lamina Dura
mDMFT-R: Modified decayed, missing, filled tooth index on radiographs
NICE: National Institute for Health Care Excellence
NICE: National Institute for Health Care Excellence
PACS: Picture Archiving and Communication System
UK: United Kingdom
